# Antiplasmodial activity of Ethanolic extract of *Cassia spectabilis* DC leaf and its inhibition effect in Heme detoxification

**DOI:** 10.1186/s12906-021-03239-9

**Published:** 2021-02-19

**Authors:** Wiwied Ekasari, Dewi Resty Basuki, Heny Arwati, Tutik Sri Wahyuni

**Affiliations:** 1 Department of Pharmacognosy and Phytochemistry, Faculty of Pharmacy, Universitas Airlangga, Campus C, Mulyorejo Street, Surabaya, 60115 Indonesia; 2 Department of Medical Parasitology, Faculty of Medicine, Universitas Airlangga, Campus A, Surabaya, 60132 Indonesia

**Keywords:** *Cassia spectabilis* DC, Combination, Heme detoxification, Plasmodium berghei, *Plasmodium falciparum*

## Abstract

**Background:**

In previous studies, *Cassia spectabilis* DC leaf has shown a good antiplasmodial activity. Therefore, this study is a follow-up study of the extract of leaf of *C. spectabilis* DC on its in vitro and in vivo antiplasmodial activity and mechanism as an antimalarial.

**Methods:**

The extract was fractionated, sub-fractionated and isolated to obtain the purified compound. In vitro antiplasmodial activity test against *Plasmodium falciparum* to find out the active compound. In vivo test against *P. berghei* ANKA-infected mice was conducted to determine prophylactic activity and antiplasmodial activity either alone or in combination with artesunate. The inhibition of heme detoxification test as one of the antimalarial mechanisms was carried out using the Basilico method.

**Results:**

The results showed that active antimalarial compound isolated from *C. spectabilis* DC leaf had a structural pattern that was identical to (−)-7-hydroxycassine. Prophylactic test of 90% ethanolic extract of *C. spectabilis* DC leaf alone against *P. berghei* ANKA*-infected* mice obtained the highest percentage inhibition was 68.61%, while positive control (doxycycline 13 mg/kg) was 73.54%. In combination with artesunate, 150 mg/kg three times a day of *C. spectabilis* DC (D_0_-D_2_) + artesunate (D_2_) was better than the standard combination of amodiaquine + artesunate where the inhibition percentages were 99.18 and 92.88%, respectively. The IC_50_ of the extract for the inhibitory activity of heme detoxification was 0.375 mg/ml which was better than chloroquine diphosphate (0.682 mg/ml).

**Conclusion:**

*C. spectabilis* DC leaf possessed potent antiplasmodial activity and may offer a potential agent for effective and affordable antimalarial phytomedicine.

## Background

Research to obtain new antimalarial drugs, both synthetic drugs and those derived from natural materials, especially from plants, is still ongoing. One of Indonesian plants that has been traditionally recognized to treat malaria is *Cassia spectabilis* DC from the Caesalpiniaceae family. Previous study related to this plant using in vivo test showed that this plant was a potential antimalarial phytomedicine [1]. New antimalarial active compounds have also been obtained from different plant, *Cassia siamea* from the same genus of *C. spectabilis* that identified as Cassiarin A alkaloid compound [2, 3]. Based on these results, an in vitro test on antiplasmodial activity to find out the active compound isolated from *C. spectabilis* DC has been conducted.

Prevention of malaria can be done in various ways, one of which is chemoprophylaxis. Chemoprophylaxis is one way to reduce the risk of malaria infection and alleviate clinical symptoms of malaria. Chemoprophylaxis used before traveling to malaria endemic area to avoid infection. In Indonesia, therapeutic choice used for malaria prophylaxis is doxycycline and tetracycline [4]. Both can be used as chemoprophylaxis for malaria but there are many undesirable effects from this drug [5]. Therefore, this research was performed to find out and develop drugs that can be used as an effective antimalarial prophylaxis, safe, have few side effects, cheap, and easy to obtain, especially those from plants, namely *C. spectabilis* DC leaf.

Furthermore, antimalarial drug development and discovery is expected to provide new drugs which is not only have antiplasmodial activity in vitro and in vivo, but also has a safety mechanism to be applied to human. Research related to the biochemical process that unique of malaria parasites plays an important role in the development of new antimalarial drugs. Malaria parasites consume hemoglobin from erythrocytes during their life cycle, however, parasites are unable to digest iron-containing heme molecule. Heme is toxic due to the reactivity of iron. Therefore, parasite has developed the mechanism to detoxify it by polymerization of heme to form hemozoin or malaria pigmen [6, 7]. The structure of hemozoin through X-ray diffraction and IR spectroscopy has been found to be similar to β-hematin [8] β-hematin is synthetic hemozoin which chemically [8], spectroscopically [9] and crystallographically [10] similar to hemozoin which consists of Ferriprotoporphyrin units linked into a polymer by propionate oxygen-iron bonds [8, 11]. The inhibition of heme detoxification has been the target of antimalarial drugs, such as chloroquine and artemisinin [12, 13], since inhibit the heme detoxification can kill the parasites. Currently, the effect of ethanol extract of *C. spectabilis* DC leaf on biochemical activity such as potential inhibition of heme detoxification in the food vacuole of malaria parasite and stage-specific activity against asexual stage of parasite has been tested.

In addition, parasitic resistance to some of the existing antimalarial drugs is the biggest problem in overcoming this disease, especially in malaria endemic areas [14]. The combination therapy with artemisinin derivatives or commonly referred to artemisinin-based combination therapy (ACT) is highly recommended by WHO as the preferred therapy that is able to control the spread of resistance of *P. falciparum* [15, 16]. Previous studies have found an effective dose of 90% ethanolic extract of *C. spectabilis* DC leaf was 150 mg/kg bodyweight which were given three times daily [1].

Based on this result, both ethanolic extract of *C. spectabilis* DC leaf alone and in combination with artesunate has been tested to determine the inhibition of growth of parasites in *P. berghei* ANKA-infected mice in vivo, since the ACT is more effective in reducing parasitemia [17]. The combination therapy will be carried out in extract-drug regimens and an overview of the resulting antimalarial activity will be obtained. A therapeutic effect of an appropriate combination of artesunate and 90% ethanolic extract of *C. spectabilis* DC leaf is reported herein.

## Methods

### Plant material

*C. spectabilis* DC leaf was obtained from Purwodadi Botanical Garden-Indonesian Institute of Sciences [Lembaga Ilmu Pengetahuan Indonesia, LIPI], Pasuruan District, East Java Province, Indonesia, and the determination of specimen was performed at the above institution. The specimen was then deposited as herbarium in the Department of Pharmacognosy and Phytochemistry, Faculty of Pharmacy, Universitas Airlangga with registration number of 02/W/XI/2016.

### Parasite and culture preparation

*Plasmodium falciparum* 3D7 strain was obtained from Faculty of Pharmacy, Universitas Airlangga, Surabaya. The parasite was cultured in complete RPMI 1640 medium supplemented with 5.96 g HEPES, 0.05 g hypoxanthine, 2.1 g NaHCO_3_, 50 μg/ml gentamycin and completed with 10% human O+ serum under anaerobe condition and incubated in a 37 °C incubator [18]. Parasitemia was observed daily prior to antimalarial assay. *Plasmodium berghei* ANKA strain was originally obtained from Eijkman Institute for Molecular Biology, Jakarta, and maintained at Faculty of Pharmacy, Universitas Airlangga. The *P. berghei* ANKA was infected into male BALB/c mice and observed the parasitemia level.

### Experimental animals

Experimental animal used in this study was BALB/c strain male mice obtained from Faculty of Veterinary Medicine of Universitas Airlangga with a weight of ±20–30 g. Mice were acclimatized for 2 weeks at a temperature of 24 ± 1 °C and humidity of 55 ± 5% prior to in vivo test. In all in vivo experiments, the animals were kept in cages with raised, wide-mesh floors to prevent coprophagy. The ethical certificate was obtained from the Ethic Commission of Faculty of Veterinary Medicine of Universitas Airlangga No: 2.KE.181.10.2018. At the end of the tests, all animals were followed the euthanasia procedure. The mice were sacrificed by cervical dislocation after anesthesia by intraperitoneal injection of 100 mg/kg bodyweight ketamine [19]. The dead animals were then buried.

### Extraction

The extract was made by three times macerating dried *C. spectabilis* DC leaf powder using 90% ethanol. The macerated extract was then evaporated using a rotavapor.

### Isolation of compound from *C. spectabilis* DC leaf

A thousand grams of dried powder of *C. spectabilis* DC leaf was macerated with n-hexane, then the pulp powder was extracted again with methanol. The extract was subjected to liquid-liquid partition using ethyl acetate and 3% tartaric acid. The isolation proceess was done by adding 3% tartaric acid until the atmosphere becomes acidic to turn the alkaloids in alkaline form into alkaloids salts. This alkaloid salt was partitioned using ethyl acetate, and the alkaloids were present in the aqueous acidic layer at the bottom. The alkaloid was the isolated from their salt form in aqueous layer, by adding NaCO_3_ to increase the pH to 9–10, and the alkaloids returned to alkaline form. Furthermore, the aqueous layer with pH 9–10 was then extracted liquid-liquid using chloroform. The fractions so called chloroform fraction.

The isolate pure alkaloid compound from other compounds, the chloroform fraction, was further fractionationed by column chromatography using stationary phase of silica gel 60 with a series of gradient mobile phases was started using 100% chlorofom, chloroform:ethyl acetate (CHCl_3_: EtOAc; 2:1), chloroform:ethyl acetate:methanol (CHCl_3_: EtOAc: MeOH; 2:1:2), and finally 100% methanol. The selection of the mobile phase was based on the results of the orientation by TLC, which gave good separation results. The solvent used for each gradient was 200 ml and the collected fractions were combined based on similarity of the TLC profile. Nine fractions (C.1-C.9) were obtained.

**Fig. 1:**
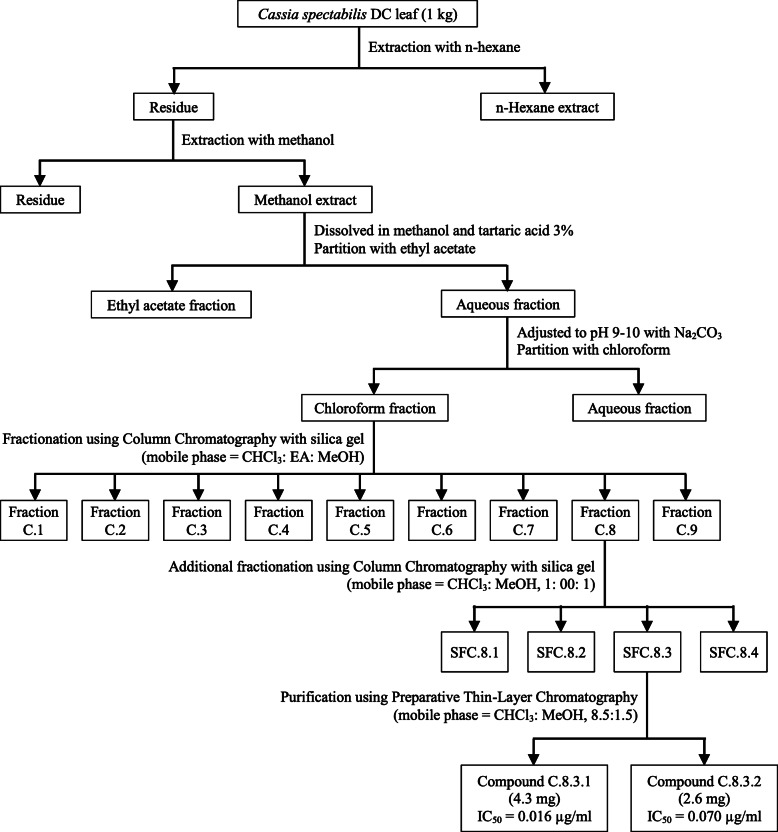
Flowchart of isolation steps of antiplasmodial alkaloid from *C. spectabilis* DC leaf

The fractions were then assayed for their antiplasmodial activity. The active fraction was then subjected to silica gel 60 column chromatography and was eluted using CHCl_3_: MeOH (1: 00: 1) to yield sub-fractions (SFC.8.1-SFC.8.4). The sub-fractions were further assayed for their antiplasmodial activity. The antimalarial active sub-fraction was then purified by preparative thin-layer chromatography (PTLC) using CHCl_3_: MeOH (8.5:1.5) to yield compounds C.8.3.1 and C.8.3.2. Identification of active compound was carried out using the TLC-densitometry, UV-Vis spectrophotometry, FTIR spectroscopy, and NMR. The procedures employed for the preparation of the plant active compounds are illustrated in Fig. 1.

### In vitro antiplasmodial activity test

The extract was dissolved in DMSO (the final DMSO concentration in a well culture plate not more than 0.5%) and diluted with complete RPMI medium containing RPMI 1640, 10% human plasma, 25 mM HEPES, and 25 mM NaHCO_3_ to make the final concentrations of 10, 1, 0.1, 0.01, and 0.001 μg/ml. Stock of parasite cultures were further diluted with uninfected type O+ human erythrocytes and culture medium to make initial parasitemia of 1% and a hematocrit of 2%. This final parasite culture was immediately used for antiplasmodial assay. The test was carried out in duplicate. The plates containing parasite cultures and extracts were then incubated in a 37 °C incubator in a candle jar for 48 h. Observation of stage-specific antiplasmodial activity in this in vitro test was performed by sampling the blood films from each well at 6, 12, 24, and 48 h. At the end of test thin films were prepared from each well and stained with 10% Giemsa solution prior to counting parasitemia [20]. The 50% inhibitory concentration (IC_50_) value was determined using probit analysis based on the relation of log concentration of test compound and % inhibition of parasites growth.

### In vivo antimalarial prophylactic activity test

In vivo test for antimalarial prophylactic activity of 90% ethanolic extract of *C. spectabilis* DC leaf used Peters method with slight modification [21]. Forty two male adult BALB/c mice were randomly divided into six groups [22]. Group 1 as a negative control group was given a 0.5% Na CMC suspension solution. Groups 2, 3, 4, and 5 were given extract at the doses of 100, 200, 400, and 800 mg/kg, respectively. Group 6 as a positive control group was given a doxycycline of 13 mg/kg suspension solution. Each treatment was given orally once a day for 4 days before parasite infection. On the fourth day the mice were infected with *P. berghei* ANKA. Each mouse in each group was infected with 1 × 10^6^ infected erythrocytes. Thin blood films from each mouse were made at 72 h post infection. Determination of parasitemia, percentage of the parasites’ growth, percentage inhibition of parasites’ growth, and effective dose 50 (ED_50_) were based on Ekasari et al. [20].

### Suppressive effect of ethanolic extract of *C. spectabilis* DC leaf combined with artesunate

The purpose of this test was to increase the effectiveness of the extract. The suppressive effect of ethanolic extract of *C. spectabilis* DC leaf in combination with artesunate, respectively against *P. berghei* ANKA infection in mice was determined using Peters’ 4-day suppression test procedure [21]. A donor mouse densely infected with parasites was anaesthetized with chloroform and the blood was collected through cardiac puncture. The presence of parasitemia was established by microscopic examination of a thin blood film. The blood was diluted with phosphate buffered saline (PBS) so that each 0.2 ml of blood contained 1 × 10^6^ *P. berghei* ANKA infected with erythrocytes. A total of 0.2 ml of diluted blood was injected intraperitoneally into 36 healthy mice. The infected animals were randomly divided into six groups those were Group A-F. The animals were treated shortly after inoculation on day zero (D_0_). Group A was given artesunate at 36.4 mg/kg, on D_0_-D_2_. Group B was given ethanolic extract of *C. spectabilis* DC leaf at 150 mg/kg (three times a day) concurrently with artesunate at 36.4 mg/kg (once a day), on D_0_-D_2_. Group C was given ethanolic extract of *C. spectabilis* DC leaf at 150 mg/kg (three times a day) on D_0_-D_2_ and artesunate at 36.4 mg/kg (once a day), on D_0_. Group D was given ethanolic extract of *C. spectabilis* DC leaf at 150 mg/kg (three times a day) on D_0_-D_2_ and artesunate at 36.4 mg/kg (once a day), on D_2_. Group E was given amodiaquine at 72.8 mg/kg concurrently with artesunate at 36.4 mg/kg, once a day, on D_0_-D_2_. Group F received 0.2 ml of 0.5% Na CMC solution as control group. Oral route was used for all administration. On day three (D_3_), thin film was made from each mouse-tail blood stained with Giemsa. Determination of parasitemia level, percentage parasites’ growth, percentage inhibition of parasites’ growth, and the ED_50_ were as described on Ekasari et al. [20].

### Heme polymerization inhibition test

The inhibition of heme polymerization test was performed based on the Basilico method [23] with slight modification in the concentration of hematin solution and the sample used. The Basilico method is an in vitro spectrophotometric microassay of heme polymerization. A 96-well U-bottomed microplates was used in this assay. The relative amounts of polymerized and unpolymerized hematin were determined using an ELISA reader. The final concentration of the extract samples ranged from 2 to 0.01 mg/ml.

A 100 μl of 1 mM hematin solution was mixed with 0.2 M NaOH and 50 μl of the test extract. A 50 μl of glacial acetic acid solution (pH 2.6) was then added to this mixture. This test was carried out at 37 °C for 24 h. The microtube was then centrifuged at 8000 rpm for 10 min, the sediment was then washed with 200 μl of DMSO three times at 8000 rpm for 10 min. The β-hematin crystalline precipitated was dissolved in 200 μl of NaOH 0.1 M to form alkaline hematin. A 100 μl of the alkaline hematin solution was transferred to 96-well microplates and the absorbance was read by ELISA reader at a wavelength of 405 nm. The effects of each test substance on β-hematin production were calculated and compared with negative controls.

## Results

### In vitro antiplasmodial activity test

All the extracts, fractions, sub-fractions, and isolated compounds from *C. spectabilis* DC leaf were continuously screened in vitro for their antiplasmodial activity against chloroquine-sensitive *P. falciparum* (3D7 strain), using microtechnique which demonstrated on the previous study [24]. The samples for these tests were hexane extract, methanolic extract, ethyl acetate fraction and chloroform fraction. The results are shown in Table 1. Antiplasmodial activity was classified based on the Gessler et al. [25], where antiplasmodial activity of extract was considered very good, moderate, and low with IC_50_ value less than 10 μg/ml, 10 to 50 μg/ml, and more than 50 μg/ml, respectively.

**Table 1:** The percentage growth inhibition of *P. falciparum* 3D7 by extract and fractions of *C. spectabillis* DC leaf

Extract/faction	Weight (g)	Concentration (μg/ml)					IC_50_ (μg/ml)
		100	10	1	0.1	0.01	
Hexane extract	118.34	0	0	0	0	0	ND
Methanolic extract	141.68	100	68.67 ± 5.19	14.36 ± 2.26	0	0	1.10
Ethyl acetate fraction	100.21	100	96.56 ± 4.86	75.47 ± 3.38	16.41 ± 3.78	0	0.41
Chloroform fraction	4.06	100	79.80 ± 1.87	56.36 ± 12.12	31.38 ± 12.58	0	0.55

*ND* Not Detected

**Fig. 2:**
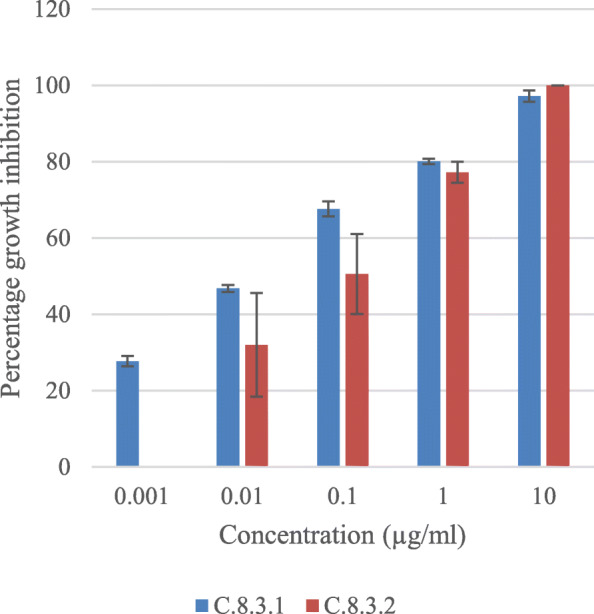
Percentage growth inhibition of *P. falciparum* 3D7 strain by compounds C.8.3.1 and C.8.3.2 of *C. spectabilis* DC leaf

During isolation of compound, chloroform was used to fractionate the extract. Chloroform fractionation resulted in nine fractions called C.1-C.9. In vitro antiplasmodial activity test of these fractions showed that fraction C.8 was the most active fraction with IC_50_ was 0.02 μg/ml (Table 2). Purification of fraction C.8 resulted in four sub-fractions called SFC.8.1-SFC.8.4, and their antiplasmodial activity is shown in Table 3. Sub-fraction SFC.8.1 and SFC.8.3 showed antiplasmodial activity against *P. falciparum* 3D7 strain with IC_50_ 0.012 and 0.015 μg/ml, respectively. The purification of sub-fractions SFC.8.3 resulted in two compounds called compound C.8.3.1 and C.8.3.2 and their antiplasmodial activity is shown in Table 4 and Fig. 2. Compound C.8.3.1 and C.8.3.2 showed antiplasmodial activity against *P. falciparum* 3D7 strain with IC_50_ 0.016 and 0.070 μg/ml, respectively.

**Table 2:** The percentage growth inhibition of *P. falciparum* 3D7 by chloroform fractions of *C. spectabillis* DC leaf

Weight of fraction (mg)		Concentration (μg/ml)					IC_50_ (μg/ml)
		10	1	0.1	0.01	0.001	
C.1	20.00	27.97 ± 5.46	0	0	0	0	> 10
C.2	4.10	0	0	0	0	0	ND
C.3	13.90	31.82 ± 3.51	6.39 ± 3.95	0	0	0	> 10
C.4	17.90	93.07 ± 6.36	59.02 ± 1.38	28.22 ± 8.39	10.54 ± 9.24	0	0.384
C.5	7.70	100	62.74 ± 6.03	24.03 ± 1.19	0	0	1–0.1
C.6	104.40	97.40 ± 3.67	93.50 ± 6.81	65.50 ± 18.95	14.92 ± 7.44	0	0.060
C.7	94.20	91.90 ± 1.23	84.84 ± 2.91	68.22 ± 4.91	48.83 ± 11.03	0	0.011
C.8	366.10	100	100	84.39 ± 7.54	66.26 ± 3.58	42.90 ± 4.50	0.002
C.9	36.50	100	87.36 ± 6.41	65.51 ± 3.78	58.52 ± 1.33	37.96 ± 15.12	0.005

*ND* Not Detected

**Table 3:** The percentage growth inhibition of *P. falciparum* 3D7 by sub-fractions of *C. spectabillis* DC leaf

Sub-faction	Weight (mg)	Concentration (μg/ml)					IC_50_ (μg/ml)
		10	1	0.1	0.01	0.001	
SFC.8.1	8.65	99.56 ± 0.59	86.42 ± 3.19	70.95 ± 0.17	62.71 ± 3.82	53.30 ± 4.67	0.001
SFC.8.2	11.25	0	0	0	0	0	0
SFC.8.3	32.50	100	72.84 ± 6.81	62.58 ± 6.01	46.59 ± 0.47	31.08 ± 7.40	0.016
SFC.8.4	20.50	76.74 ± 16.36	0	0	0	0	> 1

**Table 4:** The percentage growth inhibition of *P. falciparum* 3D7 by compounds C.8.3.1 and C.8.3.2 of *C. spectabillis* DC leaf

Compound	Weight (mg)	Concentration (μg/ml)					IC_50_ (μg/ml)
		10	1	0.1	0.01	0.001	
C.8.3.1	4.30	97.23 ± 1.49	80.11 ± 0.69	67.61 ± 1.93	46.77 ± 0.91	27.71 ± 1.35	0.016
C.8.3.2	2.60	100	77.21 ± 2.81	50.56 ± 10.48	31.96 ± 13.61	0	0.070

**Fig. 3:**
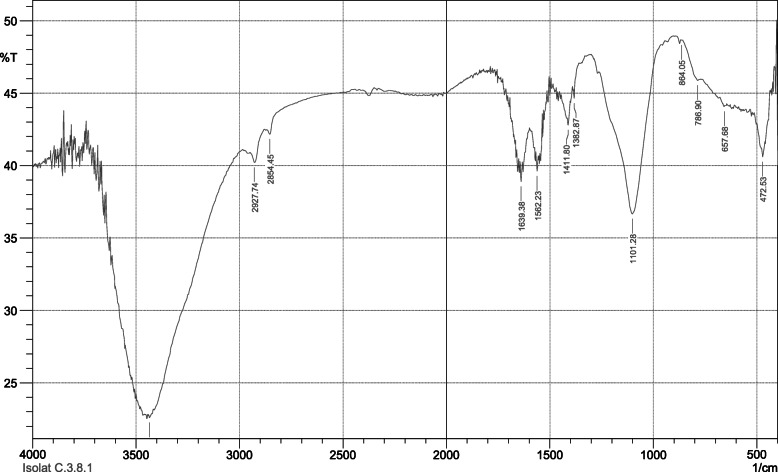
Fourier transform infra-red spectra of compound C.8.3.1 from *C. spectabilis* DC leaf

Identification using TLC-densitometry showed that compound C.8.3.1 was in the range of λ 200–300 nm, with a value of *R*_F_ = 0.65. Identification using FTIR spectroscopy showed an absorption peak at 472.53 cm^− 1^; 657.68 cm^− 1^; 786.9 cm^− 1^; 864.05 cm^− 1^; 1101.28 cm^− 1^; 1382.87 cm^− 1^ (Fig. 3).

Identification using ^1^H-NMR spectroscopy showed a characteristic signal of two hydroxyl protons at δ 3.60 ppm (1H, s); and δ 3.69 ppm (1H, s), two protons from the CH_2_ group of benzene at δ 1.91 ppm (2H, s, H-4) and δ 1.68 ppm (2H, s, H-5), one methyl group at δ 2.13 ppm (3H, s, H-12′), and some cassettes of the CH_2_ groups of the aliphatic chain at δ 1.38 ppm (2H, s, H-1′); at δ 1.22 ppm (2H, s, H-2′- H-8′); at δ 1.51 ppm (2H, s, H-9′), and at δ 2.35 ppm (2H, t, H-10′). Identification using ^13^C-NMR spectroscopy showed the presence of one carbon with a ketone group at δ 179.0 ppm, carbon in the benzene group at δ 56.96 ppm; δ 67.13 ppm; δ 29.23 ppm; δ 25.77 ppm; and δ 48.29 ppm, carbon in the aliphatic chain at δ 34.32 ppm; δ 25.87 ppm; δ 29.23 ppm; δ 29.33 ppm; δ 22.89 ppm; and δ 38.87 ppm, one carbon in the methyl group at δ 30.23 ppm, and one carbon in the hydroxyl group at δ 65.27 ppm.

### Effect of *C. spectabilis* on parasitic stage development

**Fig. 4:**
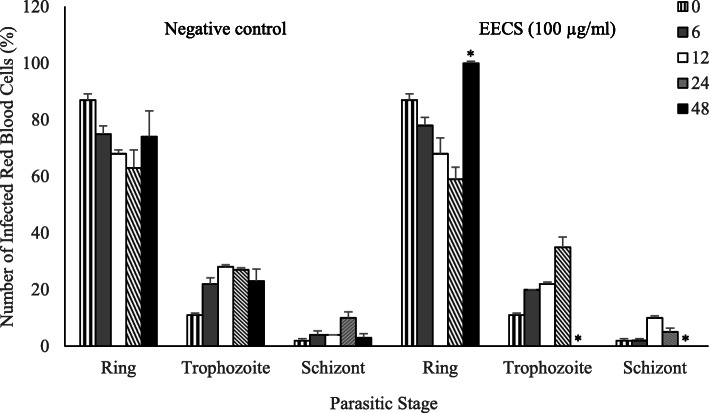
Stages development of *P. falciparum* 3D7 in vitro treated with 90% ethanolic extract of *C. spectabilis* DC leaf at different incubation time. Parasitic morphology and the development of each stage were assessed at the beginning of incubation (0 h) and at 6, 12, 24, and 48 h. The stages of parasite were classified in three groups: ring, trophozoite, and schizont. The parasitic differential count was reported as a percentage of the total red blood cells infected with those stages. Five bars in each group according to different incubation times (left to right): 0, 6, 12, 24, and 48 h. EECS = 90% ethanolic extract of *C. spectabilis* DC leaf. *p < 0.05

Stage-specific activity test of 90% ethanolic extract of *C. spectabilis* DC leaf against *P. falciparum* 3D7 strain was performed at different incubation periods of 0, 6, 12, 24, and 48 h to find out the effect of the extract to the growth of parasites at each stage of development. The extract concentration used was 100 μg/ml. The results of this experiment are shown in Table 5 and Fig. 4.

**Table 5:** Stage-specific activity of 90% ethanolic extracts of *C. spectabilis* DC leaf against *P. falciparum* 3D7 strain

Samples	Incubation time (hours)	Parasitic stage			% parasitemia	% growth	% inhibition
		Ring	Trophozoite	Schizont			
Negative control	0	41	5	1	0.76	–	–
	6	38	11	2	0.84	0.08	–
	12	36	15	2	0.86	0.10	–
	24	45	19	7	1.14	0.38	–
	48	95	30	4	2.06	1.30	–
90% ethanolic extract of *C. spectabilis* DC leaf	0	41	5	1	0.76	–	–
	6	38	10	1	0.81	0.05	37.5
	12	34	11	5	0.79	0.03	70
	24	22	13	2	0.59	0	100
	48	17	0	0	0.28	0	100

% parasitemia was obtained from total number of parasites divide to ring, trophozoite and schizont

% growth was obtained from % parasitemia an incubation time minus % parasitemia at 0 h

% inhibition was calculated according to the following formula: % inhibition = ((parasitemia in negative control – parasitemia in treatment group) / parasitemia in negative control) × 100

Negative control: The cultures were only added with DMSO, without any extract. The DMSO was used as negative control because DMSO was used to dissolve the extract

**Fig. 5:**
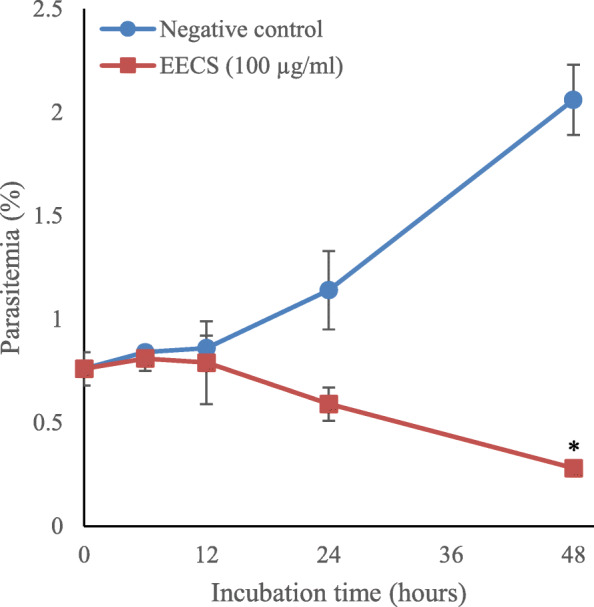
Percentage of parasitemia of 90% ethanolic extract *C. spectabilis* DC leaf and control at each incubation time against *P. falciparum* 3D7 in vitro. EECS = 90% ethanolic extract of *C. spectabilis* DC leaf. *p < 0.05

No sharp difference was observed on the growth of each parasitic stage during incubation period of 0–12 h. However, opposite direction of percentage parasitemia was seen at 12 to 48 h post incubation compared with control which moved upward but the tests extract went to 0. At 12–24 h of incubation period, parasite growth decreased but not significantly compared to negative control. Whereas at 48 h of incubation, parasite growth decreased significantly (*p* = 0.005) compared to negative control, with an inhibition percentage of 100% (Fig. 5).

### In vivo antiplasmodial prophylactic activity test

The results of in vivo antiplasmodial prophylactic activity test of 90% ethanolic extract of *C. spectabilis* DC leaf against *P. berghei* ANKA infection in mice is shown in Table 6.

**Table 6:** Prophylactic effects of 90% ethanol extract of *C. spectabilis* DC leaf to the growth of parasites compared with controls

Sample	Dose (mg/kg)	Parasitemia (%)	Inhibition (%)
90% ethanolic extract of *C. spectabilis* DC leaf	100	5.95 ± 1.21	40.14
	200	4.06 ± 1.00	59.16
	400	3.97 ± 0.89	60.06
	800	3.12 ± 0.44	68.61
Doxycycline	13	2.63 ± 0.88	73.54
Na CMC	–	9.94 ± 1.81	–

Na CMC were used as a negative control

The dose of extract of 800 mg/kg provided the greatest inhibitory effect (68.61%) compared to other doses. Probit analysis resulted in ED_50_ value was 161.20 mg/kg.

### In vivo antiplasmodial suppressive activity test

Table 7 shows the results of suppressive activity tests by *C. spectabilis* DC leaf extract combined with artesunate. Suppressive effects produced by the three extract-artesunate combinations were higher than artesunate alone. Moreover, the suppressive effects of group D (ethanolic extract of *C. spectabilis* DC leaf at 150 mg/kg (three times a day) on D_0_-D_2_ and artesunate at 36.4 mg/kg on D_2_) was higher (99.18%) than those showed by artesunate alone (82.60%) and artesunate-amodiaquine combination (92.88%).

**Table 7:** Suppressive effect of 90% ethanolic extract of *C. spectabilis* DC leaf combined with artesunate to the growth of parasite in mice

Treatment	Parasitemia count		Suppression (%)
	D_0_	D_3_	
Na CMC	1.84 ± 0.59	9.14 ± 2.38	–
*C. spectabilis* DC + Artesunate (D_0_-D_2_)	2.01 ± 0.64	3.11 ± 1.30	84.93
*C. spectabilis* DC (D_0_-D_2_) + Artesunate (D_0_)	2.92 ± 0.73	3.40 ± 1.21	90.14
*C. spectabilis* DC (D_0_-D_2_) + Artesunate (D_2_)	2.22 ± 1.14	2.08 ± 1.28	99.18
Artesunate	2.27 ± 0.76	3.40 ± 1.24	82.60
Amodiaquine + Artesunate	1.62 ± 0.74	1.97 ± 0.68	92.88

Na CMC were used as a negative control

### Heme polymerization inhibition test

The IC_50_ value of the heme inhibition test by 90% ethanolic extract of *C. spectabilis* DC leaf was 0.375 mg/ml, while chloroquine as an antimalarial standard compound was 0.682 mg/ml (Table 8).

**Table 8:** Inhibitory effect of 90% ethanolic extract of *C. spectabilis* DC leaf on the heme polymerization compared with controls

Samples	Concentration (mg/ml)	Level of hematin (mM)	Inhibition (%)	IC_50_ (mg/ml)
90% ethanolic extract of *C. spectabilis* DC leaf	2	67.32 ± 4.49	76.49 ± 1.57	0.375
	1	85.34 ± 1.42	70.20 ± 0.50	
	0.5	118.49 ± 2.92	58.62 ± 1.02	
	0.25	186.37 ± 6.55	34.91 ± 2.29	
	0.1	223.85 ± 4.35	21.82 ± 1.52	
	0.01	252.00 ± 1.85	11.99 ± 0.64	
Chloroquine diphosphate	2	99.80 ± 2.50	65.15 ± 0.97	0.682
	1	125.65 ± 4.03	56.12 ± 1.41	
	0.5	158.00 ± 4.63	44.82 ± 1.62	
	0.25	186.78 ± 3.95	34.76 ± 1.38	
	0.1	207.05 ± 1.96	27.69 ± 0.68	
	0.01	229.53 ± 9.26	19.84 ± 3.24	
DMSO	–	286.33 ± 2.92	–	–

DMSO were used as a negative control

## Discussion

**Fig. 6:**
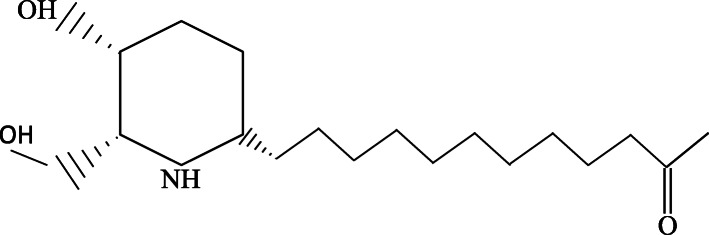
Molecular structure of (−)-7-hydroxycassine

Anti-malarial activities of *C. spectabilis* DC leaf was conducted to extract, fraction and pure isolate. The active compounds were identified by TLC-densitometry, UV-Vis spectrophotometry, FTIR spectroscopy, and NMR. In the ^1^H-NMR spectra, compound C.8.3.1 showed similarities to the compound (−)-7-hydroxycassine (Fig. 6) in the presence of several similar chemical shifts [26].

The greatest prophylactic inhibitory effect in vivo of 90% ethanolic extract of *C. spectabilis* DC leaf against *P. berghei* ANKA was 68.61% provided by the mice treated with 800 mg/kg of the extract. This result was lower than that of mice treated with doxycycline (73.54%). The highest dose that can be used in mice is 1000 mg/kg of bodyweight [27].

The effect of 90% ethanolic extract of *C. spectabilis* DC leaf to inhibit heme detoxification process showed the IC_50_ value of 0.375 mg/ml which was higher than that of chloroquine as a standard antimalarial drug which was 0.682 mg/ml.

The potential for 90% ethanolic extract of *C. spectabilis* DC leaf in the inhibition of hemozoin formation caused morphological and growth disturbances of malaria parasites due to membrane damage and disruption of the activity of several enzymes [28] as seen during the 12-h incubation period where the inhibitory activity 70% increase compared to controls. Furthermore, after 24 h incubation, the growth of parasites was 100% inhibited compared to the control (Table 5).

The advantage of the combination therapy was to increase the effectiveness of extract to prevent or slow the onset of resistance to a single antimalarial drug [29]. Selection of 90% ethanolic extract of *C. spectabilis* DC leaf combination with artesunate referred to the basis of malaria treatment which is a standard antimalarial drug recommended by WHO [30].

The dose of 90% ethanolic extract of *C. spectabilis* DC leaf used was 150 mg/kg given three times [1]. Artesunate is the artemisinin derivative is a schizonticidal with fast onset of action and gametocytocidal which can reduce malaria transmission in endemic areas [31]. Artesunate at a dose of 36.4 mg/kg was converted dose from human to mouse. The three times a day of artesunate therapy was compared to single dose therapy to prevent the immediate emerging of parasites resistance to this antimalarial drug. The control used in this study was a single dose of artesunate alone at the dose of 36.4 mg/kg and amodiaquine alone at the dose of 72.8 mg/kg which were given for 3 days. As recommended by WHO that ACT administration that given for 3 days has been able to effectively inhibit the growth of parasites, beside antimalarial drug should be effective, safe, and used in a short time. The Na CMC was used as negative control since this solvent was used to dissolve the extract in this test.

The interesting result was seen in the combination therapy using three times a day of 90% ethanolic extract of *C. spectabilis* DC leaf at dose of 150 mg/kg for 3 days with a single dose of artesunate at the dose of 36.4 mg/kg given on the third day. This combination therapy showed a higher inhibitory activity (99.18%) compared to that of combination artesunate and amodiaquine for 3 days (92.88%). This combination therapy may overcome the resistance of parasites to artesunate because shorten the duration of treatment period. Thus, the combination of 90% ethanolic extract of *C. spectabilis* DC leaf with artesunate can be expected as a new antimalarial combination drug which may replace the artesunate-amodiaquine combination drug that has been used so far. The mixture of artesunate-amodiaquine caused the drug preparations will become unstable [32]. Concurrent drug administration in a separate formula have the disadvantage of reducing patient compliance to take the drug, but the combination therapy can shorten the duration of treatment therapy [30].

## Conclusion

The results show 90% ethanolic extract of *C.spectabilis* DC leaf has a very good antiplasmodial activities both in vitro and in vivo study and revealed potentiated effect in combination with artesunate. The 90% ethanol extract of *C. spectabilis* DC leaf also found to be active in inhibiting the heme detoxification process. Compound of (−)-7-hydroxycassine may play as important role in anti-malarial activities. Therefore, this plant can be potentially used as a new source for the development of new plant-based antimalarial agent.

## Data Availability

The all data used to support the findings of this study are available from the corresponding authors upon request.

## Abbreviations

ACT: Artemisinin-based combination therapy
CHCl_3_: Chloroform
DMSO: Dimethyl sulfoxide
ED_50_: Median effective dose
EECS: Ethanolic extract of *Cassia spectabilis* DC leaf
ELISA: Enzyme-linked immunosorbent assay
EtOAc: Ethyl acetate
Fe(II)PPIX: Ferrous-protoporphyrin IX
FTIR: Fourier-transform infrared
HEPES: N-2-hydroxyethylpiperazine-N-ethanesulfonic acid
IC_50_: The half maximal inhibitory concentration
IR: Infrared
MeOH: Methanol
Na CMC: Sodium carboxymethylcellulose
NaHCO_3_: Sodium bicarbonate
NaOH: Sodium hydroxide
NMR: Nuclear magnetic resonance
PBS: Phosphate buffered saline
PTLC: Preparative thin-layer chromatography
RPMI: Roswell Park Memorial Institute
TLC: Thin-layer chromatography
UV-Vis: Ultraviolet-visible
WHO: World health organization
